# Area-Selective Atomic Layer Deposition through Selective Passivation of SiO_2_ with a SF_6_/H_2_ Plasma

**DOI:** 10.1021/acs.chemmater.4c03316

**Published:** 2025-07-11

**Authors:** Olaf C. A. Bolkenbaas, Marc J. M. Merkx, Nicholas J. Chittock, Ilker Tezsevin, Wilhelmus M. M. Kessels, Tania E. Sandoval, Adriaan J. M. Mackus

**Affiliations:** † Department of Applied Physics, 3169 Eindhoven University of Technology, P.O. Box 513, 5600 MB Eindhoven, The Netherlands; ‡ Oxford Instruments Plasma Technology, Severn Beach, Bristol BS35 4GG, U.K.; § Department of Chemical and Environmental Engineering, Universidad Técnica Federico Santa María, Av. Vicuña Mackenna 3939 Santiago, Chile

## Abstract

Area-selective atomic layer deposition (ALD) has gained widespread interest in the semiconductor industry to facilitate the continued drive for more powerful and efficient devices. In this work, we chemically passivate SiO_2_ with a single SF_6_/H_2_/Ar plasma pretreatment to selectively deposit TiO_2_ on ZnO, HfO_2_, or Al_2_O_3_, using tetrakis­(dimethylamido)­titanium (TDMAT) and H_2_O. The SF_6_/(H_2_ + SF_6_) flow ratio was tuned to suppress the etching of SiO_2_ while the nucleation delay of TiO_2_ ALD was maximized. A plasma with an SF_6_/(SF_6_ + H_2_) ratio of 0.24 etched less than 1 Å and gave the longest nucleation delay at a substrate temperature of 150 °C. After this pretreatment, 2.1, 2.0, and 1.6 nm of TiO_2_ can be deposited with a selectivity of 90% with respect to a SiO_2_ nongrowth area on HfO_2_, Al_2_O_3_, or ZnO respectively. In-situ reflection absorption infrared spectroscopy (RAIRS) measurements show that during the SF_6_/H_2_/Ar plasma, Si–OH and Si–H surface groups are replaced by Si–F groups, suggesting that full chemical passivation of SiO_2_ is achieved. The reaction of TDMAT with a F-terminated SiO_2_ surface is shown to be unfavorable using density functional theory (DFT) calculations. Furthermore, RAIRS measurements and DFT simulations show that after the plasma treatment, precursors do react on fluorinated Al_2_O_3_. Taken together, the results of this study show that using a plasma pretreatment for chemical passivation of the nongrowth area provides interesting opportunities for future ASD processes.

## Graphical abstract

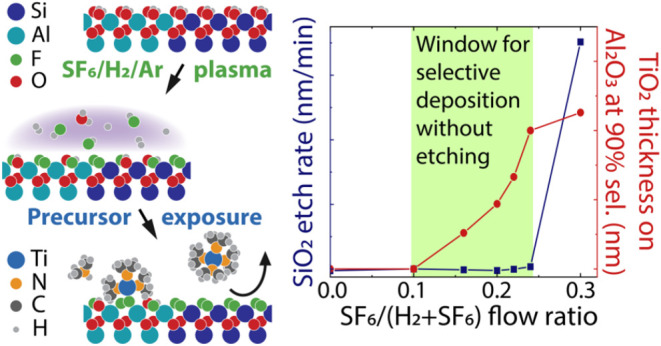

## Introduction

Over time fabrication of semiconductor devices has become more complex. Recently, this has led to an increasing interest in bottom-up fabrication approaches, allowing for a simplification of the process flows.^1–3^ One bottom-up approach is area-selective deposition (ASD),^1,2^ a collective term used to describe deposition processes of a target material on a specific surface, referred to as the growth areas, while leaving other materials, referred to as nongrowth areas, uncovered. The growth and nongrowth areas can be differentiated by chemical differences, such as different surface terminations, or by the topography of the surface.^1,2^ A deposition technique that is often considered for ASD applications is atomic layer deposition (ALD). ALD is already being used in semiconductor fabrication, for instance, one of many examples is the gate-dielectric in modern logic devices.^4^ ALD makes use of alternating self-limiting surface reactions separated by purge steps to build up a material with a very accurate thickness control, high uniformity, and conformality.^4^ The nucleation of ALD films heavily relies on the chemical surface reactions that take place during the initial cycles.^2^ In area-selective ALD, this dependence is exploited to get deposition on the growth area, while suppressing deposition on the nongrowth area. This means ASD can facilitate the transfer of a pre-existing pattern, from previous processing steps to the next layer up, with perfect self-alignment.

If the ALD chemistry is not inherently selective, inhibitor molecules or pretreatments can be used to obtain a nucleation delay on the nongrowth area.^1,2^ There are two main types of inhibition, namely self-assembled monolayers (SAMs) and small molecule inhibitors (SMIs). Both SAM monomers and SMIs consist of a reactive group that adsorbs on the nongrowth area and an inert group that passivates the surface. The SAM monomers also have a long alkane chain that helps the molecules to form a densely packed layer on the surface due to van der Waals attraction.^1,2,5^ To effectively block the ALD chemistry, inhibitors rely on two mechanisms.^6–9^ First, steric shielding, which makes use of the steric bulk of the adsorbed inhibitor to prevent precursors from reaching the reactive surface sites. Second, chemical passivation, where the SMIs chemically react with the surface groups of the nongrowth area, such that the adsorption sites for the precursor are consumed, and no precursor adsorption can take place anymore. For SMIs, both effects are required to achieve high selectivity.^6,8^ A pretreatment, like a plasma treatment or an anneal, has the potential to fully chemically passivate the surface of the nongrowth area. This simplifies the mechanisms involved in the process by eliminating the need for steric shielding.

SiO_2_ is the most common dielectric in semiconductor fabrication. Therefore, it would be relevant to have ASD processes that consider SiO_2_ as either a growth or nongrowth area. Most ASD strategies with dielectric growth and nongrowth areas consider SiO_2_ as the growth area.^1,10–12^ For a SiO_2_ nongrowth area often silanes are employed, which due to their high reactivity adsorb on many different types of surfaces.^13,14^ This work focuses on a strategy in which SiO_2_ functions as a nongrowth area, and other oxides as growth areas. This is achieved with a SF_6_/H_2_/Ar plasma pretreatment that fluorinates all dielectric surfaces and subsequently blocks deposition on SiO_2_ but not on other oxide surfaces. Previously, F-containing chemistries have been known to facilitate area-selective ALD with Si-compounds as the nongrowth area. For example, F-based precursors for W, Mo, and MoS_2_ deposition have been used for area-selective ALD.^15–19^ For these processes, the fluorination of the Si compounds by the F-containing precursors is a possible explanation for the achieved selectivity. Other examples of F-based chemistry in ASD are NF_3_ and CF_4_ plasma etching steps that have been applied as correction steps to etch back the deposited material in deposition and etch supercycles. Two examples of this include an NF_3_/O_2_ plasma for the etching of selectively deposited TiO_2_ ^20^ or Ta_2_O_5_ ^21^ and a CF_4_ plasma for the etching of SiO_2_.^12^ After these etching steps, a longer nucleation delay was observed on SiO_2_ and Si, which was beneficial for the selectivity of the entire process. The increase in the nucleation delay after the plasma exposure was attributed to fluorination of the Si and SiO_2_ surfaces. More recently, a CF_4_/N_2_ plasma etch of native SiO_2_ on SiN substrates was shown to aid the selectivity of TiO_2_ and TiON ALD using (dimethylamino)­trimethylsilane as an SMI on SiO_2_/SiN patterns.^22^ Taken together, previous literature suggests that fluorination of SiO_2_ has potential as the basis for an area-selective ALD strategy with a SiO_2_ nongrowth area.

HF is a potential fluorine source, however, the reactors used for the experiments in this work are incompatible with vapor phase HF.^23^ A different fluorine source could be SF_6_ plasmas, which have been used to fluorinate and/or etch various materials, for instance for surface modification in atomic layer etching (ALE).^24,25^ SF_6_ plasmas continuously etch materials with volatile fluorides such as Ti- and Si-based materials and would therefore etch the targeted SiO_2_ nongrowth area. Recently, Hossain et al. showed that by diluting an SF_6_ plasma with H_2_, the etching of TiO_2_ and TiN can be slowed down or fully suppressed.^23^ It is expected that H radicals scavenge F radicals in the plasma to produce HF molecules, which do not etch the substrates used in the applied conditions and are expected to act the same as vapor phase HF.^23,26,27^ Even though HF solutions are used to remove SiO_2_ layers from Si,^28–31^ HF-vapor etching requires the use of catalyst molecules due to high energy barriers for breaking Si–O–Si bridges.^32,33^ Therefore, a H_2_-diluted SF_6_ plasma, in which all F radicals are scavenged, provides a way for chemically modifying the SiO_2_ surface without etching it.

In this work, an SF_6_/H_2_/Ar plasma pretreatment was used to achieve a nucleation delay for thermal TiO_2_ ALD on SiO_2_. TiO_2_ was selected as the deposited material since it is a promising hard mask material for advancing the patterning of Si-based materials,^22,34^ and is used for self-aligned multiple patterning.^35,36^ First, the process was characterized by using in situ spectroscopic ellipsometry. After this, the passivation mechanism was investigated using in situ reflection absorption infrared spectroscopy (RAIRS) and density functional theory (DFT). Lastly, some opportunities provided by a plasma pretreatment for ASD are discussed.

## Experimental Section

### ALD Process and Sample Preparation

Thermal TiO_2_ ALD was performed using tetrakis­(dimethylamido)titanium (TDMAT) and H_2_O at 150 °C, similar to the process described in refs 37–39 . The recipe existed of 0.75 s TDMAT dosing followed by a 12 s purge, and a 0.1 s H_2_O dose with a 14 s purge followed by a 30 s ellipsometry measurement after every cycle.

The SiO_2_ substrates were coupons from a 450 nm thermal oxide wafer. The other substrates were prepared using ALD on standard Si wafers. The Al_2_O_3_ was deposited using trimethyl-aluminum (TMA) and O_2_ plasma, the HfO_2_ was prepared using tris­(dimethylamido)­cyclopentadienyl-hafnium (TDMACpHf) and an O_2_ plasma, and the ZnO was prepared using diethyl-zinc (DEZ) and H_2_O.

TDMAT (CAS number 3275–24–9) was purchased from Sigma-Aldrich, TDMACpHf (CAS number 941596–80–1) and DEZ (CAS number 557–20–0) were purchased from Dock Chemicals, and bis­(diethylamino)­silane (BDEAS) (CAS number 27804–64–4) was purchased from Strem Chemicals. All precursors were dosed using a vapor-drawn method and kept in a stainless-steel bubbler at 70, 60, 30 and 50 °C for TDMAT, TDMACpHf, DEZ and BDEAS respectively.

### In-Situ Spectroscopic Ellipsometry

A commercial Oxford Instruments FlexAL reactor was used for the in situ spectroscopic ellipsometry experiments. The reactor is equipped with an inductively coupled plasma (ICP) source and is further described in ref 40.

A J.A. Woollam M2000D ellipsometer with a photon energy range of 1.3–5 eV was used at an angle of 70° to characterize the SiO_2_ etching and TiO_2_ deposition. Both SiO_2_ and TiO_2_ were modeled using Cauchy’s equation with a refractive index of 1.46 and 2.21 at 632.8 nm, respectively. All experiments were performed at a table temperature of 150 °C. However, the substrate temperature is likely lower than the set table temperature.^41,42^

### In-Situ Reflection Absorption Infrared Spectroscopy

The in situ RAIRS experiments were performed on a home-built ALD reactor previously described in refs 6,43 . A Bruker INVENIO Fourier transform IR spectrometer with Globar IR source and a liquid N_2_ cooled MCT detector were used in combination with focusing mirrors for the RAIRS measurements. All infrared experiments were performed with a table temperature of 150 °C.

The Al_2_O_3_ and SiO_2_ surfaces for the in situ RAIRS experiments were prepared by 50 ALD cycles on an Al metal surface using TMA and BDEAS respectively and an O_2_ plasma as the coreactant.

### SF_6_ Plasma Pretreatment

All SF_6_ plasma treatments were performed using an ICP plasma source with a plasma power of 100 W, a chamber pressure of ∼50 mTorr, and a table temperature of 150 °C. On the Oxford Instruments FlexAL reactor, the plasma gas flows were controlled using MFCs, with the Ar flow always set to 150 sccm and the total SF_6_ and H_2_ flow was kept constant at 50 sccm, while the ratio between the two was varied. On the home-built setup, the gas flows were set manually using needle valves to control the partial pressures of the different gases to obtain the right ratios. In the RAIRS experiments, the Ar partial pressure was kept at 75% of the total pressure in the chamber.

### Computational Methods

The density functional theory (DFT) computations were conducted using the Vienna ab initio Simulation Package (VASP, version 5.4.4).^44–46^ The electron–ion interactions were modeled using the projector-augmented wave (PAW) approach.^47,48^ The study utilized the Perdew–Burke–Ernzerhof (PBE) exchange-correlation functional within the generalized gradient approximation (GGA), enhanced by the D3 dispersion correction and the Becke–Johnson (BJ) damping function.^49–51^ The plane-wave basis set was defined with a kinetic energy limit of 400 eV. For structural optimizations, the force on each atom was required to be less than 0.01 eV/Å. Gaussian smearing of 0.01 eV was used throughout the study. The self-consistent-field cycle’s convergence was determined by a threshold of 10^–5^ eV. For the bulk crystalline SiO_2_, an 11 × 11 × 9 *k*-point mesh centered at Γ was automatically generated for Brillouin zone integration. Surface calculations were performed using a Γ centered 2 × 2 × 1 Monkhorst–Pack grid.^52^ The lattice parameters of the α quartz phase of SiO_2_ with P3121 space group (space group number = 152) were calculated as *a* = 4.80 Å and *c* = 5.32 Å in line with the experimental values of *a* = 4.91 Å and *c* = 5.40 Å.^53^ A four-layer (SiO_2_ layers) SiO_2_ (0001) surface was modeled as a 3 × 3 supercell of the cleaved optimized SiO_2_ bulk structure. The bottom two (SiO_2_) layers were kept frozen at their bulk positions. Hydroxylation of the SiO_2_ surface was performed based on previous studies.^7,10,54,55^ OH terminated SiO_2_ surface was terminated by 18 OH groups (2 OH terminations per Si),^56^ whereas the OH groups were replaced by F atoms to represent the SF_6_ pretreated surface (see eq 1 below). The periodicity of the slab in the direction perpendicular to the metal surface was avoided by adding a vacuum spacing of 17 Å.

To study Al_2_O_3_ surfaces, an automatically generated Γ centered 11 × 11 × 5 *k*-point mesh was used to integrate the Brillouin zone of crystalline Al_2_O_3_ bulk, whereas a Γ centered 2 × 2 × 1 Monkhorst–Pack grid was used for the surface calculations.^52^ A 4-layer 3 × 3 supercell of α-Al_2_O_3_(0001) was modeled following the same procedure as in our previous works.^6,9,57,58^ A vacuum spacing of 15 Å was added to avoid periodicity of the slab in the direction perpendicular to the surface. The resultant supercell has the *a*, *b*, *c* lattice dimensions of 14.21 Å, 14.21 Å, and 26 Å, respectively with θ = 120°. The Al_2_O_3_ surface is partially hydroxylated by locating hydroxyl groups on all 9 top layer Al atoms to achieve a hydroxylated Al_2_O_3_ (0001) surface. This resulted in a 1 monolayer OH coverage (corresponding to 5.1 OH nm^–2^), which is slightly less than the experimentally observed value of 7.1 OH nm^–2^.^59^ Nine out of the 18 exposed O atoms neighboring the top layer Al atoms are hydrogenated in order to balance the negative charge from the addition of OH. The bottom two Al_2_O_3_ layers were kept frozen at their bulk positions. Simulations on an Al-terminated surface (bare-Al_2_O_3_) were also performed. The structure used for these simulations is the same as above, prior to the hydroxylation reactions (OH to Al and H to O sites).^60,61^

It is expected that the HF species formed in SF_6_ plasma are responsible for the fluorination of the surfaces. Therefore, the feasibility of the fluorination was calculated with respect to HF molecules. The energy of the HF molecule was computed via spin-relaxed calculations in 20 Å cubic cells at the Γ point. The fluorination energy of the whole surface is calculated in eq 1, as

1 $${\Delta E}_{\mathrm{fluorination}}=\left(E_{{\mathrm{MO}}_{x}‐F‐\mathrm{term}}+{a\times E}_{H_{2}O}\right)-\left(E_{{\mathrm{MO}}_{x}‐\mathrm{OH}‐\mathrm{term}}+{a\times E}_{\mathrm{HF}}\right)$$

where MO_*x*_ refers to either SiO_2_ or Al_2_O_3_, and *a* is the number of OH groups present on the surface, which are 18 and 9 for SiO_2_ and Al_2_O_3_ respectively. Multiple physisorption and chemisorption scenarios were tested for the adsorption of the TDMAT precursor on the OH- and F-terminated SiO_2_ and Al_2_O_3_ surfaces, whereas only the energetically most favorable outputs were used to represent different reaction pathways. For the energy calculations, similar to HF, the energy of the TDMAT molecule was computed via spin-relaxed calculations in 20 Å cubic cells at the Γ point. Then, the adsorption energies were computed as

$${\Delta E}_{\mathrm{ads}}=\sum E_{\mathrm{products}}-\left(E_{{\mathrm{MO}}_{x}-F-\mathrm{term}}+E_{\mathrm{TDMAT}}\right)$$

The DFT calculations for predicting the IR vibrations were performed in Jaguar (Rapid ab initio electronic structure package of Schrödinger).^62^ B3LYP-D3 functionals were used with CSDZ**pp basis sets available in Jaguar.

## Results and Discussion

### SF_6_/H_2_/Ar Plasma for Area-Selective TiO_2_ ALD without Substrate Etching

Figure 1 shows nucleation curves for thermal TiO_2_ ALD on HfO_2_, Al_2_O_3_, ZnO, and SiO_2_ after exposure to a 10 min plasma treatment with a SF_6_/(H_2_ + SF_6_) ratio of 0.24 at 150 °C, as measured with in situ ellipsometry. No deposition is observed for the first 30 cycles on a SiO_2_ substrate, whereas immediate growth can be seen on HfO_2_ and Al_2_O_3_. 2.1 and 2.0 nm of TiO_2_ can be deposited with 90% selectivity on HfO_2_ and Al_2_O_3_ respectively, compared to SiO_2_. On ZnO, TiO_2_ has a nucleation delay of approximately 4 cycles after the plasma exposure. The amount of TiO_2_ that can be deposited with 90% selectivity on ZnO is therefore slightly lower than on HfO_2_ and Al_2_O_3_ at 1.6 nm.

**Figure 1:**
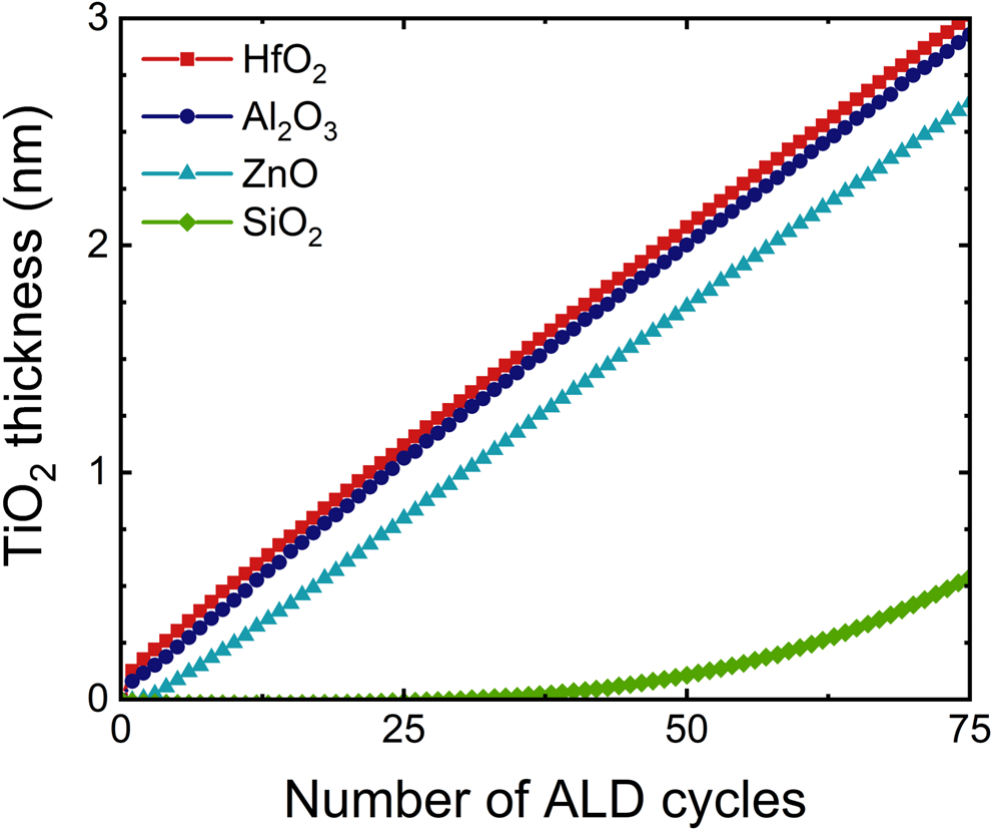
Nucleation curves of TiO_2_ ALD on SiO_2_, Al_2_O_3_, and HfO_2_ after a 10 min plasma exposure with an SF_6_/(H_2_ + SF_6_) ratio of 0.24, as measured using in situ ellipsometry.

The plasma composition and pretreatment time both affect the selectivity. To investigate the etching of the SiO_2_ nongrowth area, SiO_2_ samples were exposed to 10 min plasmas with different SF_6_/(H_2_ + SF_6_) ratios. Figure 2A shows the thickness measured after every minute of plasma exposure. No etching can be observed for SF_6_/(H_2_ + SF_6_) ratios below 0.24. At a flow ratio of 0.24, less than 1 Å was etched in 10 min. For a flow ratio of 0.30, an etch rate of 0.7 nm/min is observed after an initial onset of the continuous etching. The etch rates for different flow ratios calculated from linear fits are shown in Figure 2B. The etch rate data demonstrates the suppression of etching by dilution of the SF_6_ with H_2_, similar to what was previously observed on TiO_2_ and TiN.^23^ Based on these observations, we can conclude that below the flow ratio of 0.24, all F is scavenged from the plasma, fully suppressing the etching of the substrate.

**Figure 2:**
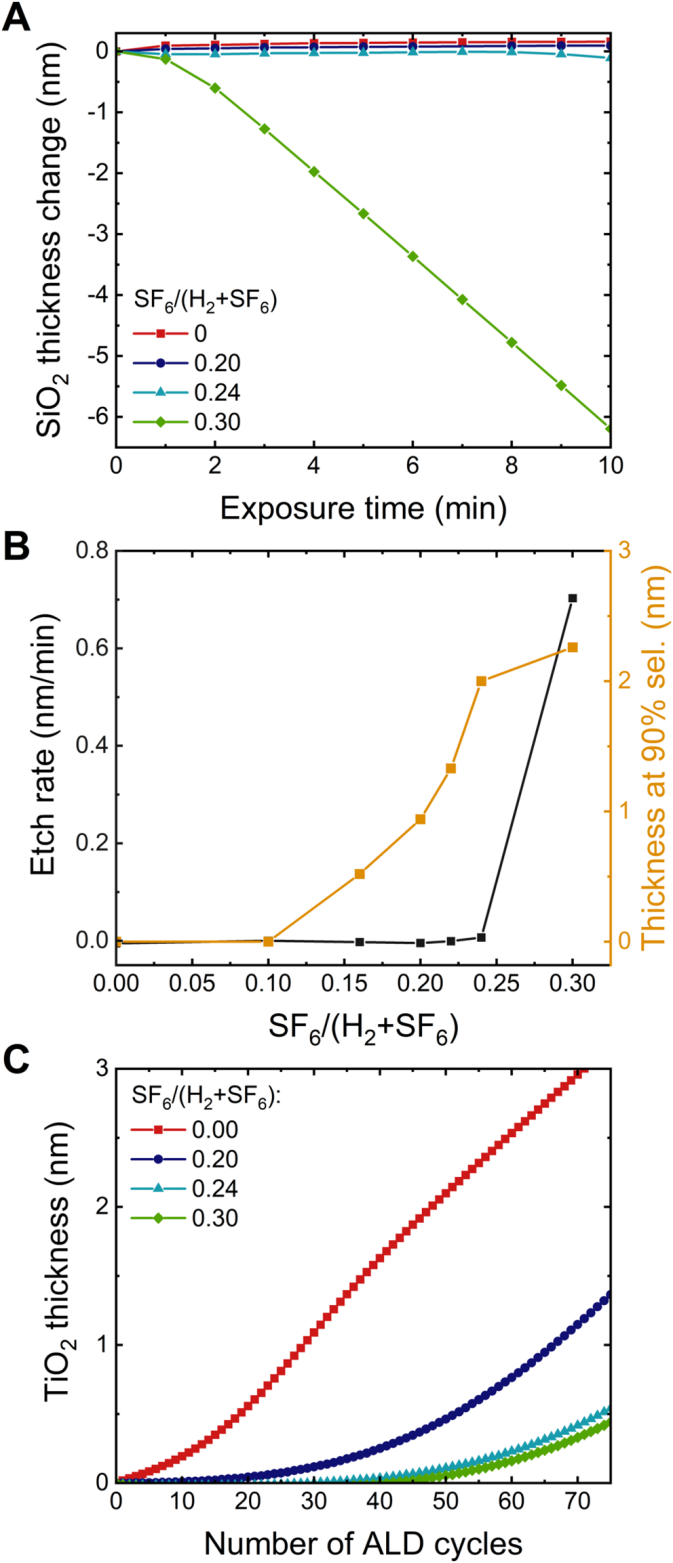
(A) SiO_2_ thickness change as a function of plasma exposure time for different SF_6_/(H_2_ + SF_6_) flow ratios, as measured using in situ spectroscopic ellipsometry. (B) The etch rate as a function of SF_6_/(H_2_ + SF_6_) flow ratio, calculated using a linear fit of the SiO_2_ thickness excluding the first 3 min of plasma exposure. In addition, TiO_2_ thickness at 90% selectivity with respect to an Al_2_O_3_ growth area is shown as a function of SF_6_/(H_2_ + SF_6_) ratio, based on the nucleation curves in C and Figure S1. (C) Nucleation curves of TiO_2_ after a 10 min plasma treatment on SiO_2_ for different SF_6_/(H_2_ + SF_6_) ratios, as measured using in situ spectroscopic ellipsometry.

The nucleation delay is also influenced by the flow ratio, as shown by the nucleation curves in Figure 2C. The in situ ellipsometry data shows that a higher SF_6_/(H_2_ + SF_6_) ratio leads to a longer nucleation delay. The TiO_2_ thickness that can be deposited on Al_2_O_3_ with 90% selectivity is also plotted in Figure 2B. This figure shows that between the flow ratios of 0.1 and 0.24, there is a window for selective deposition without etching of the SiO_2_ nongrowth area. The selectivity window likely starts at a ratio of 0.1 due to an increase in HF generation in the plasma as more SF_6_ is added. For flow rates above 0.24, the F radicals are no longer all scavenged by the H radicals, leading to etching of the SiO_2_ surface. When varying the exposure time at the flow ratio of 0.24, the longest nucleation delay was observed for plasma times ≥10 min (Figure S2). Therefore, this flow ratio and plasma time were selected for further experiments. X-ray photoelectron spectroscopy after the plasma exposure (Figure S3) shows the presence of F on the surface. Based on this and the data shown in Figure 2, it is expected that for a 10 min plasma with a flow ratio of 0.24, a maximum F concentration is obtained on the SiO_2_ surface.

### Mechanism of Inhibition

The passivation mechanism was investigated using in situ RAIRS. The spectrum taken after a SF_6_/H_2_/Ar plasma in Figure 3A shows negative peaks for both OH and Si–H at 3740 and 2210 cm^–1^ respectively, indicating that these surface groups are removed during the plasma exposure. At lower wavenumbers, several features are visible in the spectrum. The peaks at 1100 and 1010 cm^–1^ in Figure 3A closely match the predicted locations for Si–F vibrations of SiF, SiF_2_ and SiF_3_ species as calculated using DFT. Furthermore, the peak present at 935 cm^–1^ was previously reported in literature for SiF_2_ and is close to a peak calculated using DFT.^63–65^ The assignment of all three peaks is documented in more detail in Table S1 in the Supporting Information. The other features present in the 900–1250 cm^–1^ range of Figure 3A, are observed for both a SF_6_/H_2_/Ar and a pure Ar plasma treatment, and are likely caused by a shift in the SiO_2_ phonon peaks toward higher wavenumbers,^66–69^ which has been reported to occur as a result of densification.^64,70^ Plasma-enhanced ALD literature shows that densification of SiO_2_ by ion impact already occurs when the substrate is grounded.^71^ Therefore, it is likely that the densification of the SiO_2_ is caused by Ar ion impact during the plasma treatment. Taken together, the presence of Si–F and the removal of both Si–OH and Si–H suggests that the SiO_2_ surface is F-terminated after the plasma.

**Figure 3:**
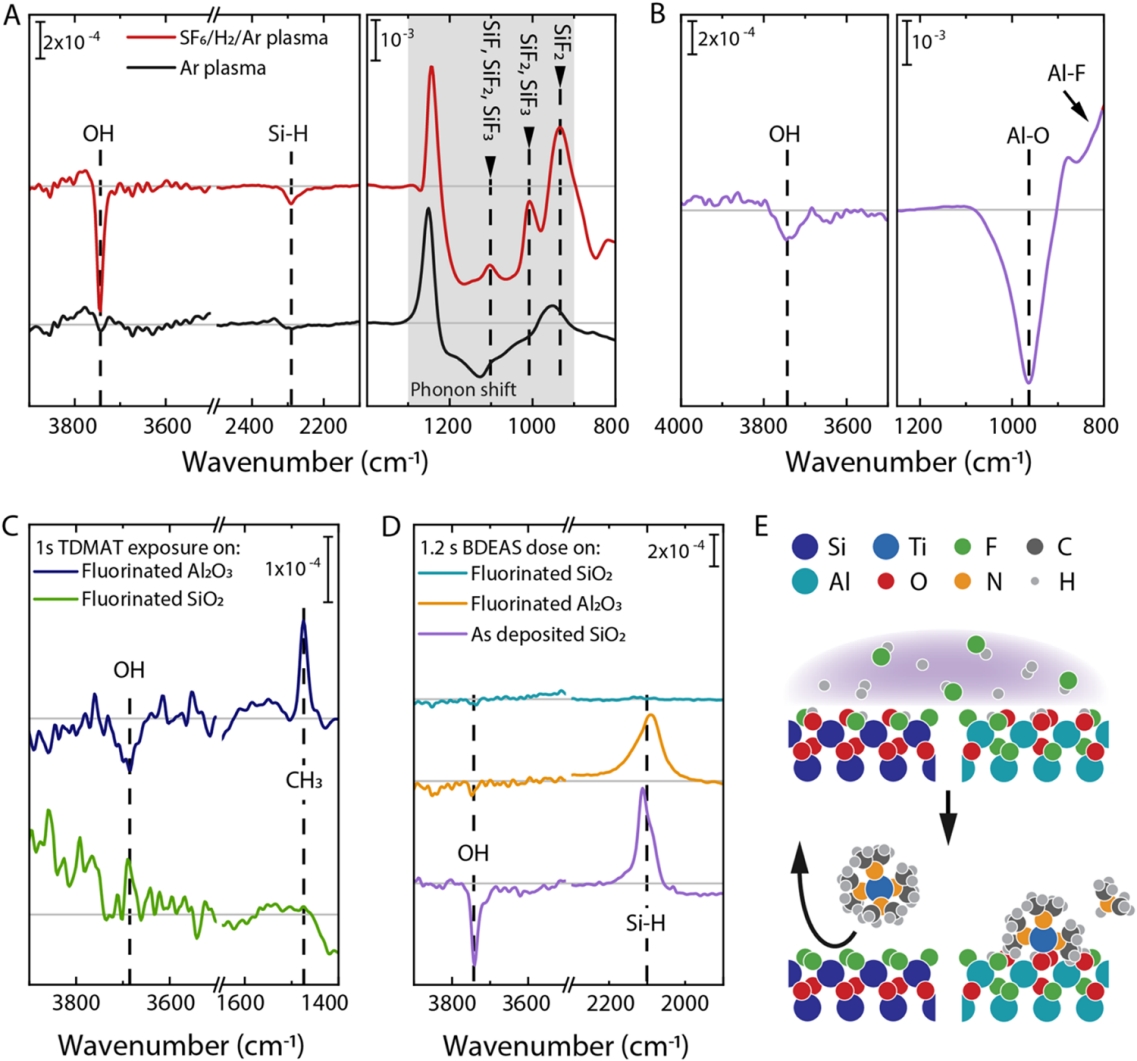
(A) In-situ RAIRS difference spectra of SiO_2_ exposed to an Ar plasma for 11 min or a 10 min SF_6_/H_2_/Ar plasma with a SF_6_/(SF_6_ + H_2_) ratio of ± 0.23. (B) Difference spectrum of Al_2_O_3_ exposed to a SF_6_/H_2_/Ar plasma. The spectra in A and B are all referenced to the spectrum before plasma exposure. (C) Difference spectra after 1 s TDMAT exposure on pretreated Al_2_O_3_ and SiO_2_, using the spectrum after plasma exposure as the reference spectrum. (D) Difference spectra after 1.2 s BDEAS exposure on fluorinated Al_2_O_3_ and SiO_2_ and as deposited SiO_2_, using the spectrum after plasma exposure as the reference spectrum. (E) A schematic representation of the proposed passivation mechanism.

To quantify the number of OH groups removed by the plasma, the OH-peak measured after a SF_6_/H_2_/Ar plasma pretreatment was compared to the OH peak after BDEAS adsorption on an as-prepared SiO_2_ surface. The negative peak area after a 10 min plasma exposure was found to be 17.3 ± 0.8% larger than the peak area after BDEAS exposure (Figure S4). From ALD literature we know that 2.5 ± 0.1 BDEAS molecules adsorb per nm^2^ at 150 °C.^72^ To obtain an estimate of how many OH groups are removed during the plasma, the assumption was made that on average 1.5 ± 0.3 OH groups are removed per adsorbed precursor molecule, since we know that each BDEAS molecule reacts with either 1 or 2 surface OH groups.^72^ Using this information and the relative peak sizes from RAIRS, the removed OH density is estimated to be 4.4 ± 0.9 OH groups per nm^2^. This value closely matches the maximum OH surface density of amorphous SiO_2_ as determined by Zhuravlev of 4.9 ± 0.5 OH/nm^2^.^73^ Therefore, even though quantification with RAIRS is challenging, it is at least plausible to assume that this ASD strategy removes all OH groups from the SiO_2_ nongrowth area, therefore achieving full chemical passivation on SiO_2_ surfaces. In contrast, in the RAIRS spectrum shown in Figure 3B, only a small negative OH peak can be observed on Al_2_O_3_ after the SF_6_/H_2_/Ar plasma treatment, together with the conversion of the Al_2_O_3_ phonon peak to AlF_3_ indicated by the peaks at low wavenumbers.^74,75^

After TDMAT exposure, see Figure 3C, the IR spectra show no peaks related to precursor adsorption on fluorinated SiO_2_ surfaces, whereas a CH_3_ peak can be observed for TDMAT exposure on pretreated Al_2_O_3_ substrates. This confirms that the precursor is blocked on the SiO_2_ after the plasma pretreatment and adsorbs on Al_2_O_3_. Furthermore, the spectra show no features related to OH bonds on SiO_2_, whereas a small amount of OH consumption can be observed on the Al_2_O_3_ surface. The data therefore indicate that not all OH groups are removed during the F-based plasma exposure on Al_2_O_3_, leaving adsorption sites for the precursor. Together the RAIRS measurements discussed above provide experimental proof that selective passivation of SiO_2_ is achieved through fluorination of the top surface.

To test whether the area-selective deposition strategy could also be used for other ALD chemistries, BDEAS adsorption on fluorinated SiO_2_ was investigated using RAIRS. The data reported in Figure 3D shows that, similarly to the spectra after TDMAT exposure, no precursor adsorption was observed on the fluorinated SiO_2_ surface, whereas clear peaks indicating precursor adsorption and OH removal can be seen on the pretreated Al_2_O_3_ and on the as-deposited SiO_2_. The fact that neither precursor adsorbs on the fluorinated SiO_2_ shows the versatility of the approach and the potential for the selective deposition of materials other than the TiO_2_ shown in this work. Furthermore, comparing spectra after BDEAS exposure on as-deposited and plasma-pretreated Al_2_O_3_ shows that the Si–H peak from the precursor ligand after plasma exposure is 0.71 ± 0.01 times the size of the same peak without the plasma exposure, whereas the same ratio for the OH peak is 0.20 ± 0.01 (Figure S5). Therefore, not all precursors that adsorb on the fluorinated Al_2_O_3_ react with OH groups, indicating that the precursor can also adsorb on Al–F surface groups. Figure 3E shows a schematic representation of the expected passivation mechanism based on the RAIRS data. The figure shows complete fluorination of SiO_2_ and only partial fluorination of Al_2_O_3_ surfaces during the plasma treatment, followed by selective adsorption of a precursor molecule on the Al_2_O_3_.

To further obtain an understanding of the selectivity, DFT simulations on SiO_2_ and Al_2_O_3_ surfaces were performed. The results of these calculations for SiO_2_ are summarized in Figure 4. By exchanging the OH groups on the surface with F atoms using HF (resulting in H_2_O as a reaction product), a net energy gain of 0.15 eV per surface group was calculated (Δ*E*_Rxn_ = −2.67 eV). This result confirms that it is energetically favorable for the surface OH to be replaced with F. DFT simulations of Al_2_O_3_, shown in Figure S6, also confirmed that in the presence of HF, fluorination of the surface is energetically favorable. As explained above, the RAIRS data show the removal of both Al–OH and Al–O bonds. Therefore, OH- and Al-terminated Al_2_O_3_ slabs were used for simulations, and both resulted in the same F-terminated surface. Moreover, based on ALE literature, the diffusion of F into Al_2_O_3_ is a known mechanism at these conditions, which in combination with fluorination on oxygen bridges, is the most likely cause for the loss of Al–O–Al bonds.^24^

**Figure 4:**
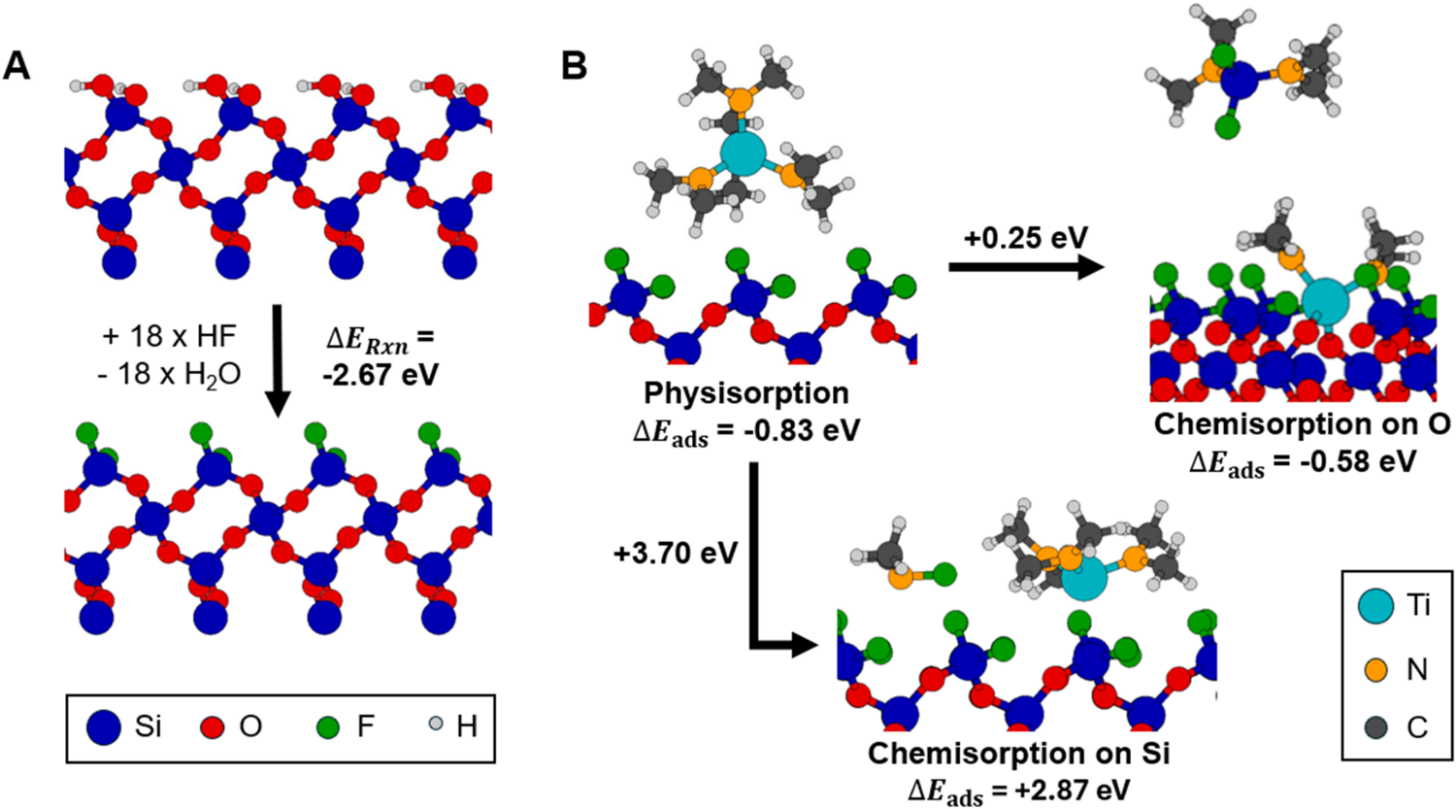
(A) Replacement of surface OH by F groups on SiO_2_, using HF as a reactant and forming H_2_O as a reaction product for all 18 OH groups present on the SiO_2_ slab, as calculated using DFT. The reaction energy Δ*E*_Rxn_ represents the total energy change for the replacement of all OH surface groups on the SiO_2_ slab. (B) Two chemisorption pathways for TDMAT on fluorinated SiO_2_.

Adsorption reactions of TDMAT on the fluorinated SiO_2_ were also studied using DFT. Figure 4B shows two potential reaction mechanisms, both of which are unlikely to take place. The simplest reaction, in which a ligand is removed from the precursor and reacts with a surface F atom such that the Ti atom bonds to the surface Si, is very unfavorable with a reaction energy of +3.70 eV. The other potential reaction between TDMAT and the fluorinated SiO_2_ etches Si from the surface after the precursor has donated two ligands to a surface Si atom. A molecule consisting of a Si atom with two F ligands and two dimethylamine ligands is formed as the reaction product, and a Ti atom with its two remaining ligands adsorbs in its place. With a reaction energy of +0.25 eV, this reaction pathway is energetically possible with the used experimental parameters, but due to the steric bulk of the precursor ligands, the kinetic barrier for this reaction is expected to be too high.^76,77^ Therefore, the results presented in this work suggest that precursors do not chemisorb on fluorinated SiO_2_, providing a pathway for achieving selectivity, similar to the expected mechanism for inhibition during and after W ALD using the WF_6_ precursor.^15,18,19^

Additional DFT simulations were carried out to understand if there is a driving force for the precursor to adsorb on fluorinated Al_2_O_3_. The physisorption energies of TDMAT on hydroxylated and fluorinated Al_2_O_3_ are summarized in Table S2. These results show that TDMAT physisorption is more thermodynamically favorable on fluorinated Al_2_O_3_ compared to fluorinated SiO_2_ with an adsorption energy of −1.85 eV versus −0.83 eV. This difference corresponds to a higher reactivity of the Al–F surface and a shorter Ti–F distance on fluorinated Al_2_O_3_ compared to fluorinated SiO_2_. Moreover, by deconvoluting the adsorption energy into electronic and dispersive contributions (Δ*E*_ads_ = Δ*E*_ads_^disp^ + Δ*E*_ads_^elec^), distinct differences are observed in the electronic component across both surfaces. This is in correspondence with a lower reactivity of F atoms in the Si–F surface in comparison to Al–F surface, due to shorter bond length and decrease in electron density.^85^ Moreover, in the case of SiO_2_–F, the surface is more sterically hindered by the higher number of F atoms per surface atom (2 per Si atom) as well as by the shorter bond length (1.58 and 1.59 Å long for the top and bottom bonds). This decreases the precursor-surface interaction compared to Al_2_O_3_–F, and thus, the electronic contribution to the adsorption energy. In the case of Al_2_O_3_–F, the Al–F sites (1 F per Al atom) bonds are 1.67 Å and less sterically hindered. Overall, the RAIRS and DFT results conclusively show that the plasma fluorinates both SiO_2_ and Al_2_O_3_ surfaces and that the F-termination of the SiO_2_ is responsible for blocking the ALD chemistry, whereas it does not have the same blocking effect on Al_2_O_3_.

Based on RAIRS measurements and DFT calculations, two different mechanisms for the loss of selectivity are expected to occur. First, DFT shows that physisorption of TDMAT on fluorinated SiO_2_ is favorable with an adsorption energy of −0.83 eV, which can cause the molecule to stick to the surface and promote nucleation.^78^ This is further supported by the nucleation curves in Figure S7, which show a shorter nucleation delay when a shorter TDMAT purge time is used. Second, Figure S8 shows that TDMAT exposure after extensive H_2_O exposure on a fluorinated surface exhibits a negative OH peak and detectable CH_3_ stretch peaks between 2705 and 3000 cm^–1^. These peaks indicate that OH groups were formed during the water dosing on which the TDMAT could then adsorb. Further TDMAT dosing shows additional adsorption of the precursor without reduction in the OH concentration on the surface, suggesting the presence of the physisorbed precursor state. This is confirmed by the presence of a slight negative CH_3_ stretch peak after 10 min of pumping after the final dose, indicating desorption of physisorbed TDMAT species. It should be noted that another mechanism for the loss of selectivity could be the decomposition of the precursor, since TDMAT has previously been found to decompose at temperatures as low as 140 °C.^79^ However, as mentioned in the Experimental Section the sample temperature is lower than the set table temperature, and therefore we expect that the sample surface is sufficiently cold to prevent the decomposition of TDMAT.^41,42^ Furthermore, other literature suggests decomposition of TDMAT only becomes important at temperatures above 180 °C.^80,81^

### Opportunities Provided by Halogenation of SiO_2_

Recent work has shown that Cl- and Br-terminated Si(100) and Si(111) exhibit a nucleation delay for thermal TiO_2_ ALD using TiCl_4_ or TDMAT and H_2_O.^82,83^ Therefore, it is likely that a SiO_2_ surface terminated with a different halogen also provides opportunities for a SiO_2_ nongrowth area. This could be especially relevant when considering that SF_6_ is known to be an extremely potent greenhouse gas,^84^ making it desirable to find different halogen sources for plasmas that inhibit growth on SiO_2_ without etching. A potential replacement for SF_6_ that results in a F-terminated surface could be NF_3_ which has a global warming potential approximately a third lower than that of SF_6_ over a time span of 100 years.^84^

As in any area-selective deposition process, selectivity is lost at some point during processing. Based on the above discussion, it is expected that the physisorption of the precursor is the major cause of the loss of selectivity. The selectivity could be improved by introducing etch steps in a supercycle approach, similar to area-selective TiO_2_ and Ta_2_O_3_ processes developed by Vallat et al.^20,21^ In the process described here, the reapplication of the SF_6_/H_2_/Ar plasma could be used to both etch back the deposited TiO_2_ from the SiO_2_ and to recover the F-termination on SiO_2_ to further increase the selectivity of the process. Furthermore, the fluorination of the SiO_2_ without etching of the layer provides an opportunity in atomic layer etching as a modification step, providing a starting point for a new ALE process.^24,25^

## Conclusions

A strategy of obtaining area-selective ALD through the fluorination of SiO_2_ was successfully demonstrated. In-situ ellipsometry showed that the composition of an SF_6_/H_2_/Ar plasma could be tuned such that the SiO_2_ was not etched but caused a nucleation delay for TiO_2_ ALD. The selective deposition of TiO_2_ on fluorinated Al_2_O_3_, HfO_2_, and ZnO compared to SiO_2_ was then demonstrated and showed similar linear growth on Al_2_O_3_ and HfO_2_, whereas a 4 cycles nucleation delay was observed on ZnO. DFT and RAIRS were used to obtain a fundamental understanding of the mechanisms involved in achieving the selective adsorption of precursors between SiO_2_ and Al_2_O_3_. A chemically passivated SiO_2_ surface was created during the plasma by the replacement of OH-groups with F. On Al_2_O_3_, not all surface OH-groups were removed. The ALD precursor subsequently did not adsorb on the F-terminated SiO_2_, while it did adsorb on the Al_2_O_3_, providing a pathway for achieving selective deposition. The strategy of replacing reactive surface groups with unreactive ones for full chemical passivation of SiO_2_ shows promise for future ASD schemes.

## Supplementary Material



## Data Availability

The data that support the findings of this study are openly available in 4TU.ResearchData with reference 10.4121/e96c7616-f34a-4e39-a3ea-e78db832f8b4.

## Notes

Nucleation curves after pretreatments with different SF_6_/(H_2_ + SF_6_) flow ratios, and different plasma times; identification of IR peaks in SiO_2_ spectrum after plasma treatments; X-ray photoelectron spectra of SiO_2_ after plasma pretreatment; quantification of OH consumption on SiO_2_ during the plasma treatment; comparison of precursor and OH peak areas on Al_2_O_3_ with and without the plasma treatment; DFT results related to fluorination of Al_2_O_3_ and of TDMAT physisorption on OH and F terminated Al_2_O_3_ and SiO_2_; nucleation curves for different TDMAT purge times; and reflection absorption infrared spectra of extensive precursor and coreactant dosing (PDF)

## References

[1] Parsons G. N., Clark R. D. (2020). Area-Selective Deposition: Fundamentals, Applications,
and Future Outlook. Chem. Mater..

[2] Mackus A. J. M., Merkx M. J. M., Kessels W. M. M. (2019). From the Bottom-Up: Toward Area-Selective
Atomic Layer Deposition with High Selectivity. Chem. Mater..

[3] IEEE . International Roadmap for Devices and Systems, Lithography & Patterning 2023 https://irds.ieee.org/editions/2023/irds%E2%84%A2-2023-lithography. (accessed May 21, 2024).

[4] Knoops, H. C. M. ; Potts, S. E. ; Bol, A. A. ; Kessels, W. M. M. Atomic Layer Deposition. In Handbook of Crystal Growth: Thin Films and Epitaxy, 2nd ed.; Elsevier Inc., 2015; Vol. 3, pp 1101–1134.

[5] Yarbrough J., Shearer A. B., Bent S. F. (2021). Next Generation Nanopatterning Using
Small Molecule Inhibitors for Area-Selective Atomic Layer Deposition. J. Vac. Sci. Technol., A.

[6] Merkx M. J. M., Sandoval T. E., Hausmann D. M., Kessels W. M. M., Mackus A. J. M. (2020). Mechanism
of Precursor Blocking by Acetylacetone Inhibitor Molecules during
Area-Selective Atomic Layer Deposition of SiO_2_. Chem. Mater..

[7] Tezsevin I., Maas J. F. W., Merkx M. J. M., Lengers R., Kessels W. M. M., Sandoval T. E., Mackus A. J. M. (2023). Computational
Investigation of Precursor
Blocking during Area-Selective Atomic Layer Deposition Using Aniline
as a Small-Molecule Inhibitor. Langmuir.

[8] Yu P., Merkx M. J. M., Tezsevin I., Lemaire P. C., Hausmann D. M., Sandoval T. E., Kessels W. M. M., Mackus A. J. M. (2024). Blocking Mechanisms
in Area-Selective ALD by Small Molecule Inhibitors of Different Sizes:
Steric Shielding versus Chemical Passivation. Appl. Surf. Sci..

[9] Li J., Tezsevin I., Merkx M. J. M., Maas J. F. W., Kessels W. M. M., Sandoval T. E., Mackus A. J. M. (2022). Packing of Inhibitor Molecules during
Area-Selective Atomic Layer Deposition Studied Using Random Sequential
Adsorption Simulations. J. Vac. Sci. Technol.,
A.

[10] Mameli A., Merkx M. J. M., Karasulu B., Roozeboom F., Kessels W. (Erwin) M.
M., Mackus A. J. M. (2017). Area-Selective
Atomic Layer Deposition of SiO_2_ Using Acetylacetone as
a Chemoselective Inhibitor in an ABC-Type Cycle. ACS Nano.

[11] Liu T. L., Bent S. F. (2021). Area-Selective
Atomic Layer Deposition on Chemically
Similar Materials: Achieving Selectivity on Oxide/Oxide Patterns. Chem. Mater..

[12] Karasulu B., Roozeboom F., Mameli A. (2023). High-Throughput Area-Selective
Spatial
Atomic Layer Deposition of SiO_2_ with Interleaved Small
Molecule Inhibitors and Integrated Back-Etch Correction for Low Defectivity. Adv. Mater..

[13] Ulman A. (1996). Formation
and Structure of Self-Assembled Monolayers. Chem. Rev..

[14] Sharma R. K., Bandichhor R., Mishra V., Sharma S., Yadav S., Mehta S., Arora B., Rana P., Dutta S., Solanki K. (2023). Advanced Metal Oxide-Based Nanocatalysts for the Oxidative
Synthesis of Fine Chemicals. Mater. Adv..

[15] Oh H., Thelven J. M., Margavio H. R. M., Parsons G. N. (2024). Low-Temperature
Dual-Material Area-Selective Deposition: Molybdenum Hexafluoride-Mediated
SiO_2_ Fluorination/Passivation for Self-Aligned Molybdenum/Metal
Oxide Nanoribbons. Adv. Funct Mater..

[16] Oh H., Kim J. S., Margavio H. R. M., Parsons G. N. (2023). Self-Aligned Nanopatterning
and Controlled Lateral Growth by Dual-Material Orthogonal Area-Selective
Deposition of Poly­(3,4-Ethylenedioxythiophene) and Tungsten. Chem. Mater..

[17] Soares J., Jen W., Hues J. D., Lysne D., Wensel J., Hues S. M., Graugnard E. (2023). Intrinsic
and Atomic Layer Etching Enhanced Area-Selective
Atomic Layer Deposition of Molybdenum Disulfide Thin Films. J. Vac. Sci. Technol., A.

[18] Lemaire P. C., King M., Parsons G. N. (2017). Understanding
Inherent Substrate
Selectivity during Atomic Layer Deposition: Effect of Surface Preparation,
Hydroxyl Density, and Metal Oxide Composition on Nucleation Mechanisms
during Tungsten ALD. J. Chem. Phys..

[19] Kalanyan B., Lemaire P. C., Atanasov S. E., Ritz M. J., Parsons G. N. (2016). Using Hydrogen
to Expand the Inherent Substrate Selectivity Window during Tungsten
Atomic Layer Deposition. Chem. Mater..

[20] Vallat R., Gassilloud R., Salicio O., El Hajjam K., Molas G., Pelissier B., Vallée C. (2019). Area Selective
Deposition of TiO_2_ by Intercalation of Plasma Etching Cycles
in PEALD Process: A Bottom up Approach for the Simplification of 3D
Integration Scheme. J. Vac. Sci. Technol., A.

[21] Vallat R., Gassilloud R., Eychenne B., Vallée C. (2017). Selective
Deposition of Ta_2_O_5_ by Adding Plasma Etching
Super-Cycles in Plasma Enhanced Atomic Layer Deposition Steps. J. Vac. Sci. Technol., A.

[22] Mameli A., Tapily K., Shen J., Roozeboom F., Lu M., O’Meara D., Semproni S. P., Chen J. R., Clark R., Leusink G., Clendenning S. (2024). Unfolding
an Elusive Area-Selective Deposition Process: Atomic Layer Deposition
of TiO_2_ and TiON on SiN vs SiO_2_. ACS Appl. Mater. Interfaces.

[23] Hossain A. A., Wang H., Catherall D. S., Leung M., Knoops H. C. M., Renzas J. R., Minnich A. J. (2023). Isotropic
Plasma-Thermal Atomic Layer
Etching of Superconducting Titanium Nitride Films Using Sequential
Exposures of Molecular Oxygen and SF_6_/H_2_ Plasma. J. Vac. Sci. Technol., A.

[24] Chittock N. J., Vos M. F. J., Faraz T., Kessels W. M. M., Knoops H. C. M., Mackus A. J. M. (2020). Isotropic Plasma
Atomic Layer Etching of Al_2_O_3_ Using a Fluorine
Containing Plasma and Al­(CH_3_)_3_. Appl. Phys. Lett..

[25] Chittock N. J., Shu Y., Elliott S. D., Knoops H. C. M., Kessels W. M. M., Mackus A. J. M. (2023). Isotropic
Atomic Layer Etching of GaN Using SF_6_ Plasma and Al­(CH_3_)_3_. J. Appl. Phys..

[26] Pankratiev P. A., Barsukov Y. V., Kobelev A. A., Vinogradov A. Y., Miroshnikov I. V., Smirnov A. S. (2020). Etching of Si_3_N_4_ by SF_6_/H_2_ and SF_6_/D_2_ Plasmas. J. Phys. Conf Ser..

[27] Catherall D. S., Hossain A. A., Ardizzi A. J., Minnich A. J. (2024). Atomic Layer Etching
of SiO_2_ Using Sequential Exposures of Al­(CH_3_)_3_ and H_2_/SF_6_ Plasma. J. Vac. Sci. Technol., A.

[28] Ubara H., Imura T., Hiraki A. (1984). Formation
of Si-H Bonds on the Surface
of Microcrystalline Silicon Covered with SiO_x_, by HF Treatment. Solid State Commun..

[29] Yablonovitch E., Allara D. L., Chang C. C., Gmitter T., Bright T. B. (1986). Unusually
Low Surface-Recombination Velocity on Silicon and Germanium Surfaces. Phys. Rev. Lett..

[30] Burrows V. A., Chabal Y. J., Higashi G. S., Raghavachari K., Christman S. B. (1988). Infrared Spectroscopy of Si(111) Surfaces after HF
Treatment: Hydrogen Termination and Surface Morphology. Appl. Phys. Lett..

[31] Higashi G. S., Chabal Y. J., Trucks G. W., Raghavachari K. (1990). Ideal Hydrogen
Termination of the Si(111) Surface. Appl. Phys.
Lett..

[32] Kang J. K., Musgrave C. B. (2002). The Mechanism of HF/H_2_O Chemical Etching
of SiO_2_. J. Chem. Phys..

[33] Jang W. I., Choi C. A., Lee M. L., Jun C. H., Kim Y. T. (2002). Fabrication
of MEMS Devices by Using Anhydrous HF Gas-Phase Etching with Alcoholic
Vapor. J. Micromech. Microeng..

[34] Nye R. A., Van Dongen K., De Simone D., Oka H., Parsons G. N., Delabie A. (2023). Enhancing
Performance and Function of Polymethacrylate
Extreme Ultraviolet Resists Using Area-Selective Deposition. Chem. Mater..

[35] Dallorto S., Staaks D., Schwartzberg A., Yang X., Lee K. Y., Rangelow I. W., Cabrini S., Olynick D. L. (2018). Atomic Layer Deposition
for Spacer Defined Double Patterning of Sub-10 Nm Titanium Dioxide
Features. Nanotechnology.

[36] Raley, A. ; Thibaut, S. ; Mohanty, N. ; Subhadeep, K. ; Nakamura, S. ; Ko, A. ; O’Meara, D. ; Tapily, K. ; Consiglio, S. ; Biolsi, P. In Self-Aligned Quadruple Patterning Integration Using Spacer on Spacer Pitch Splitting at the Resist Level for Sub-32nm Pitch Applications, SPIE Proceedings, Lin, Q. ; Engelmann, S. U. , Eds.; SPIE, 2016.

[37] Xie Q., Jiang Y. L., Detavernier C., Deduytsche D., Van Meirhaeghe R. L., Ru G. P., Li B. Z., Qu X. P. (2007). Atomic
Layer Deposition of TiO_2_ from Tetrakis-Dimethyl-Amido Titanium
or Ti Isopropoxide Precursors and H_2_O. J. Appl. Phys..

[38] Maeng W. J., Kim H. (2006). Thermal and Plasma-Enhanced ALD of Ta and Ti Oxide Thin Films from
Alkylamide Precursors. Electrochem. Solid-State
Lett..

[39] Katamreddy R., Omarjee V., Feist B., Dussarrat C. (2008). Ti Source
Precursors for Atomic Layer Deposition of TiO_2_, STO and
BST. ECS Trans..

[40] Heil S. B. S., van Hemmen J. L., Hodson C. J., Singh N., Klootwijk J. H., Roozeboom F., van de Sanden M. C. M., Kessels W. M. M. (2007). Deposition of
TiN and HfO_2_ in a Commercial 200mm Remote Plasma Atomic
Layer Deposition Reactor. J. Vac. Sci. Technol.,
A.

[41] Knoops H. C. M., Braeken E. M. J., de Peuter K., Potts S. E., Haukka S., Pore V., Kessels W. M. M. (2015). Atomic
Layer Deposition of Silicon Nitride from Bis­(Tert-Butylamino)­Silane
and N_2_ Plasma. ACS Appl. Mater. Interfaces.

[42] Clemente, I. E. ; Miakonkikh, A. V. In Application of Spectral Ellipsometry to in Situ Diagnostics of Atomic Layer Deposition of Dielectrics on Silicon and AlGaN, SPIE Proceedings, Lukichev, V. F. ; Rudenko, K. V. , Eds.; SPIE, 2016.

[43] Merkx M. J.
M., Tezsevin I., Yu P., Janssen T., Heinemans R. H. G. M., Lengers R. J., Chen J. R., Jezewski C. J., Clendenning S. B., Kessels W. M. M., Sandoval T. E., Mackus A. J. M. (2024). In Situ Formation
of Inhibitor Species through Catalytic Surface Reactions during Area-Selective
Atomic Layer Deposition of TaN. J. Chem. Phys..

[44] Kresse G., Furthmüller J. (1996). Efficiency
of Ab-Initio Total Energy Calculations for
Metals and Semiconductors Using a Plane-Wave Basis Set. Comput. Mater. Sci..

[45] Kresse G., Furthmüller J. (1996). Efficient Iterative Schemes for Ab
Initio Total-Energy
Calculations Using a Plane-Wave Basis Set. Phys.
Rev. B.

[46] Kresse G., Hafner J. (1993). Ab Initio Molecular
Dynamics for Open-Shell Transition
Metals. Phys. Rev. B.

[47] Blöchl P. E. (1994). Projector
Augmented-Wave Method. Phys. Rev. B.

[48] Kresse G., Joubert D. (1999). From Ultrasoft Pseudopotentials
to the Projector Augmented-Wave
Method. Phys. Rev. B.

[49] Perdew J. P., Burke K., Ernzerhof M. (1996). Generalized
Gradient Approximation
Made Simple. Phys. Rev. Lett..

[50] Grimme S., Ehrlich S., Goerigk L. (2011). Effect of
the Damping Function in
Dispersion Corrected Density Functional Theory. J. Comput. Chem..

[51] Grimme S., Antony J., Ehrlich S., Krieg H. (2010). A Consistent and Accurate
Ab Initio Parametrization of Density Functional Dispersion Correction
(DFT-D) for the 94 Elements H-Pu. J. Chem. Phys..

[52] Monkhorst H. J., Pack J. D. (1976). Special Points for
Brillonin-Zone Integrations. Phys. Rev. B.

[53] Hammond, C. R. C. R. Properties of the Elements and Inorganic Compounds. In CRC Handbook of Chemistry and Physics; Haynes, W. M. ; Lide, D. R. ; B, T. J. , Eds.; CRC Press: Boca Raton, 2014; pp 4–150.

[54] Huang L., Han B., Han B., Derecskei-Kovacs A., Xiao M., Lei X., O’Neill M. L., Pearlstein R. M., Chandra H., Cheng H. (2013). First-Principles
Study of a Full Cycle of Atomic Layer Deposition of SiO_2_ Thin Films with Di­(Sec-Butylamino)­Silane and Ozone. J. Phys. Chem. C.

[55] Li J., Wu J., Wu C. Z., Han B., Karwacki E. J., Xiao M., Lei X., Cheng H. (2009). On the Dissociative Chemisorption of Tris­(Dimethylamino)­Silane
on Hydroxylated SiO_2_(001) Surface. J. Phys. Chem. C.

[56] Huang L., Han B., Han B., Derecskei-Kovacs A., Xiao M., Lei X., O’Neill M. L., Pearlstein R. M., Chandra H., Cheng H. (2013). First-Principles
Study of a Full Cycle of Atomic Layer Deposition of SiO_2_ Thin Films with Di­(*Sec*-Butylamino)­Silane and Ozone. J. Phys. Chem. C.

[57] Merkx M. J. M., Jongen R. G. J., Mameli A., Lemaire P. C., Sharma K., Hausmann D. M., Kessels W. M. M., Mackus A. J. M. (2021). Insight into
the Removal and Reapplication of Small Inhibitor Molecules during
Area-Selective Atomic Layer Deposition of SiO_2_. J. Vac. Sci. Technol., A.

[58] Chittock N. J., Maas J. F. W., Tezsevin I., Merkx M. J. M., Knoops H. C. M., Kessels W. M. M. E., Mackus A. J. M. (2025). Investigation
of the Atomic Layer Etching Mechanism for Al_2_O_3_ Using Hexafluoroacetylacetone and H_2_ Plasma. J. Mater. Chem. C.

[59] Puurunen R. L., Lindblad M., Root A., Outi I Krause A. (2001). Successive
Reactions of Gaseous Trimethylaluminium and Ammonia on Porous Alumina. Phys. Chem. Chem. Phys..

[60] Elliott S. D., Greer J. C. (2004). Simulating the Atomic Layer Deposition of Alumina from
First Principles. J. Mater. Chem..

[61] Ranea V. A., Carmichael I., Schneider W. F. (2009). DFT Investigation of Intermediate
Steps in the Hydrolysis of α-Al_2_O_3_ (0001). J. Phys. Chem. C.

[62] Bochevarov A. D., Harder E., Hughes T. F., Greenwood J. R., Braden D. A., Philipp D. M., Rinaldo D., Halls M. D., Zhang J., Friesner R. A. (2013). Jaguar: A High-performance
Quantum
Chemistry Software Program with Strengths in Life and Materials Sciences. Int. J. Quantum Chem..

[63] Yoo Sik., Swope W., Sparks R., Mordo B. (1997). D. Comparison of C_2_F_6_ and FASi-4 as Fluorine
Dopant Sources in Plasma
Enhanced Chemical Vapor Deposited Fluorinated Silica Glass Films. J. Mater. Res..

[64] Campostrini R., Ischia M., Carturan G., Armelao L. (2002). Sol-Gel Synthesis and
Pyrolysis Study of Oxyfluoride Silica Gels. J. Sol-Gel Sci. Technol..

[65] Lataste E., Demourgues A., Leclerc H., Goupil J. M., Vimont A., Durand E., Labrugère C., Benalla H., Tressaud A. (2008). Access to
Highly Fluorinated Silica by Direct F_2_ Fluorination: Chemical
Compositions and FTIR Investigations. J. Phys.
Chem. C.

[66] Coblentz Society, I. ; Linstrom, P. J. ; M, W. G. Evaluated Infrared Reference Spectra. In NIST Chemistry WebBook, NIST Standard Reference Database Number 69; NIST: Gaithersburg MD, 2023.

[67] Galeener F.
L., Geissberger A. E. (1983). Vibrational
Dynamics in ^30^Si-Substituted
Vitreaous SiO_2_. Phys. Rev. B.

[68] Galeener F. L., Mikkelsen J. C. (1981). Vibrational
Dynamics in ^18^O-Substituted
Vitreous SiO_2_. Phys. Rev. B.

[69] Hwang S. M., Kim H. S., Le D. N., Sahota A., Lee J., Jung Y. C., Kim S. W., Kim S. J., Choi R., Ahn J., Hwang B. K., Zhou X., Kim J. (2022). High Wet-Etch Resistance
SiO_2_ Films Deposited by Plasma-Enhanced Atomic Layer Deposition
with 1,1,1-Tris­(Dimethylamino)­Disilane. J. Vac.
Sci. Technol., A.

[70] Han S. M., Aydil E. S. (1997). Detection of Combinative Infrared Absorption Bands
in Thin Silicon Dioxide Films. Appl. Phys. Lett..

[71] Arts K., Deijkers J. H., Faraz T., Puurunen R. L., Kessels W. M. M. E., Knoops H. C. M. (2020). Evidence for
Low-Energy Ions Influencing Plasma-Assisted
Atomic Layer Deposition of SiO_2_: Impact on the Growth per
Cycle and Wet Etch Rate. Appl. Phys. Lett..

[72] Dingemans G., van Helvoirt C. A. A., Pierreux D., Keuning W., Kessels W. M. M. (2012). Plasma-Assisted
ALD for the Conformal Deposition of SiO_2_: Process, Material
and Electronic Properties. J. Electrochem. Soc..

[73] Zhuravlev L. T. (2000). The Surface
Chemistry of Amorphous Silica. Zhuravlev Model. Colloids Surf., A.

[74] Vos M. F. J., Knoops H. C. M., Kessels W. M. M., Mackus A. J. M. (2021). Reaction
Mechanisms
during Atomic Layer Deposition of AlF_3_ Using Al­(CH_3_)_3_ and SF_6_ Plasma. J. Phys. Chem. C.

[75] DuMont J. W., George S. M. (2017). Competition between
Al_2_O_3_ Atomic
Layer Etching and AlF_3_ Atomic Layer Deposition Using Sequential
Exposures of Trimethylaluminum and Hydrogen Fluoride. J. Chem. Phys..

[76] Oh I.-K., Sandoval T. E., Liu T.-L., Richey N. E., Nguyen C. T., Gu B., Lee H.-B.-R., Tonner-Zech R., Bent S. F. (2022). Elucidating the
Reaction Mechanism of Atomic Layer Deposition of Al_2_O_3_ with a Series of Al­(CH_3_)_
*x*
_Cl_3–*x*
_ and Al­(C_
*y*
_H_2*y*+1_)_3_ Precursors. J. Am. Chem. Soc..

[77] Park B.-E., Oh I.-K., Lee C. W., Lee G., Shin Y.-H., Lansalot-Matras C., Noh W., Kim H., Lee H.-B.-R. (2016). Effects
of Cl-Based Ligand Structures on Atomic Layer Deposited HfO_2_. J. Phys. Chem. C.

[85] Wang H., Wu P., Wu Z., Shi L., Cheng L. (2022). New insight into the
electronic structure of SiF_4_: synergistic back-donation
and the eighteen-electron rule. Phys. Chem.
Chem. Phys..

[78] Oh I.-K., Sandoval T. E., Liu T.-L., Richey N. E., Bent S. F. (2021). Role of
Precursor Choice on Area-Selective Atomic Layer Deposition. Chem. Mater..

[79] Sønsteby H. H., Yanguas-Gil A., Elam J. W. (2020). Consistency and Reproducibility in
Atomic Layer Deposition. J. Vac. Sci. Technol.,
A.

[80] Elam J. W., Schuisky M., Ferguson J. D., George S. M. (2003). Surface Chemistry
and Film Growth during TiN Atomic Layer Deposition Using TDMAT and
NH_3_. Thin Solid Films.

[81] Musschoot J., Xie Q., Deduytsche D., Van den Berghe S., Van Meirhaeghe R. L., Detavernier C. (2009). Atomic Layer Deposition of Titanium Nitride from TDMAT
Precursor. Microelectron. Eng..

[82] Parke T., Silva-Quinones D., Wang G. T., Teplyakov A. V. (2023). The Effect
of Surface Terminations on the Initial Stages of TiO_2_ Deposition
on Functionalized Silicon. ChemPhysChem.

[83] Mason J. R., Teplyakov A. V. (2024). Preparation
of Br-Terminated Si(100) and Si(111) Surfaces
and Their Use as Atomic Layer Deposition Resists. J. Vac. Sci. Technol., A.

[84] Forster, P. ; Ramaswamy, V. ; Artaxo, P. ; Berntsen, T. ; Betts, R. ; Fahey, D. W. ; Haywood, J. ; Lean, J. ; Lowe, D. C. ; Myhre, G. ; Nganga, J. ; Prinn, R. ; Raga, G. ; Schulz, M. ; Van Dorland, R. Changes in Atmospheric Constituents and in Radiative Forcing. In Climate Change 2007: The Physical Science Basis.; Solomon, S. ; Qin, D. ; Manning, M. ; Chen, Z. ; Marquis, M. ; Averyt, K. B. ; Tignor, M. ; Miller, H. L. , Eds.; Contribution of Working Group I to the Fourth Assessment Report of the Intergovernmental Panel on Climate Change; Cambridge University Press: Cambridge, 2007.

