# Supplementary material for: Area-Selective Atomic Layer Deposition through Selective Passivation of SiO2 with a SF6/H2 Plasma
Source: Chem Mater. 2025 Jul 11;37(14):4982–91. doi: 10.1021/acs.chemmater.4c03316 (PMC12287997; doi:10.1021/acs.chemmater.4c03316)
Supplement: Supplementary file 1 [file cm4c03316_si_001.pdf]

Supporting information:

Area-selective Atomic Layer Deposition through Selective Passivation of SiO_2 with a SF_6/H_2 Plasma

Olaf C.A. Bolkenbaas[1], Marc J.M. Merkx[1], Nicholas J. Chittock[1,2], Ilker Tezsevin[1], Wilhelmus M.M. Kessels[1], Tania E. Sandoval[3,], Adriaan J.M. Mackus[1,*]*

[1] Department of Applied Physics, Eindhoven University of Technology, P.O. Box 513, 5600 MB Eindhoven, The Netherlands

[2] Oxford Instruments Plasma Technology, Severn Beach, Bristol, BS35 4GG, UK

[3] Department of Chemical and Environmental Engineering, Universidad Técnica Federico Santa María, Av. Vicuña Mackenna 3939, Santiago, Chile

* Email: a.j.m.mackus@tue.nl
* Email: tania.sandoval@usm.cl

Nucleation curves for different flow ratios:

To compare the selectivity for different $SF_6/(H_2+SF_6)$ flow ratios, the selectivity (S) was quantified using the thicknesses on SiO_2 (t_{NGA}), and on Al_2O_3 (t_{GA}) measured after each cycle and using the equation for selectivity[1,2]

$$S = \frac{t_{GA} - t_{NGA}}{t_{GA} + t_{NGA}}$$

By calculating the selectivity per cycle using the data shown in Figure S1, the thickness on the growth area could be calculated for $S = 0.9$. These thicknesses were reported in Figure 2B.

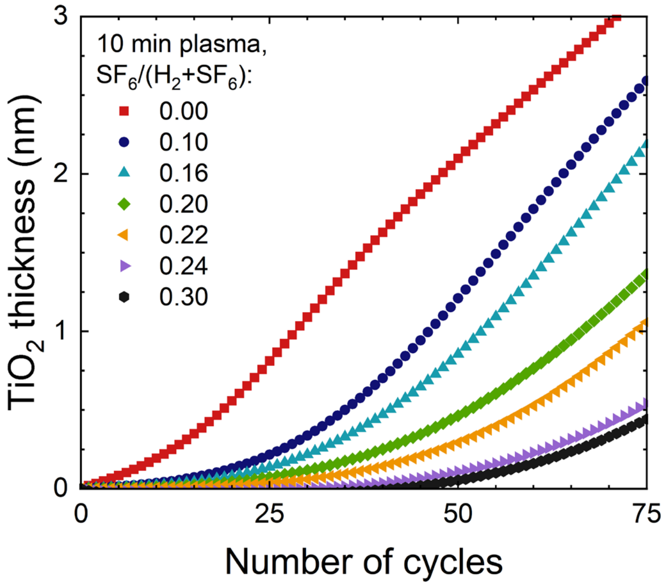

Figure S1: Nucleation curves of TiO_2 after a 10 minute plasma treatment on SiO_2 for different $SF_6/(H_2+SF_6)$ ratios, as measured using in-situ spectroscopic ellipsometry.

Influence of plasma exposure time on the nucleation delay:

The plasma exposure time was varied to find the plasma exposure time that gave the highest selectivity for an $SF_6/(H_2+SF_6)$ flow ratio of 0.24. The nucleation curves in Figure S2 show that the optimum time is 10 minutes.

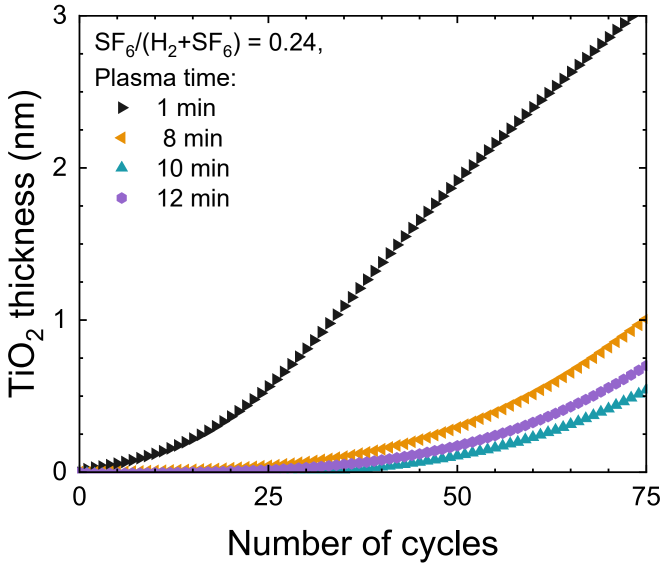

Figure S2: Nucleation curves of TiO_2 after plasma treatment with different durations on SiO_2 with an $SF_6/(H_2+SF_6)$ flow ratio of 0.24.

Silicon fluoride peak identification:

Both literature and density functional theory (DFT) modeling have been used to identify peaks related to Si-F vibrations. The DFT calculations were performed on $Si(OH)_xF_{4-x}$ clusters, based on the general approach used in references [3,4]. The calculated peak locations had to be shifted by a factor of 1.05 so that the peak at the highest wavenumber matched in both the experiments and simulations at 1100 cm^{-1}. Table S1 shows the identified peaks for different $Si(OH)_xF_{4-x}$ molecules matched to experimentally determined peaks visible in Figure 3A. The DFT calculations suggest a peak at 900 cm^{-1}, but this peak was experimentally measured (closer to the value reported in literature of 935 cm^{-1} [5–7]) at 930 cm^{-1}, which is in line with small deviations between theory and experiments based on anharmonicity effects.

Table S1: Silicon fluoride peak identification based on experiments, DFT simulations and literature.

**Simulation wavenumber (cm^{-1})**	**Experimental wavenumber (cm^{-1})**	**Species**	**Vibration**	**Literature band [source]**
1100	1100	O_3-SiF, O_2-SiF_2, O-SiF_3	Asymmetric O-Si-F stretch	
1030	1010	O_2-SiF_2	Asymmetric Si-F stretch	
1010	1010	O_3-SiF	Asymmetric O-Si stretch	
900	930	O_2-SiF_2	O-Si-O scissoring	935 cm^{-1} [5–7]

X-ray photoelectron spectroscopy of SiO_2 after the pretreatment:

X-ray photoelectron spectroscopy of an SiO_2 surface after exposure to a $SF_6/H_2/Ar$ plasma clearly showed the presence of F and a lack of S on the surface (the rise in counts around 159 eV is caused by a Si2s plasmon).

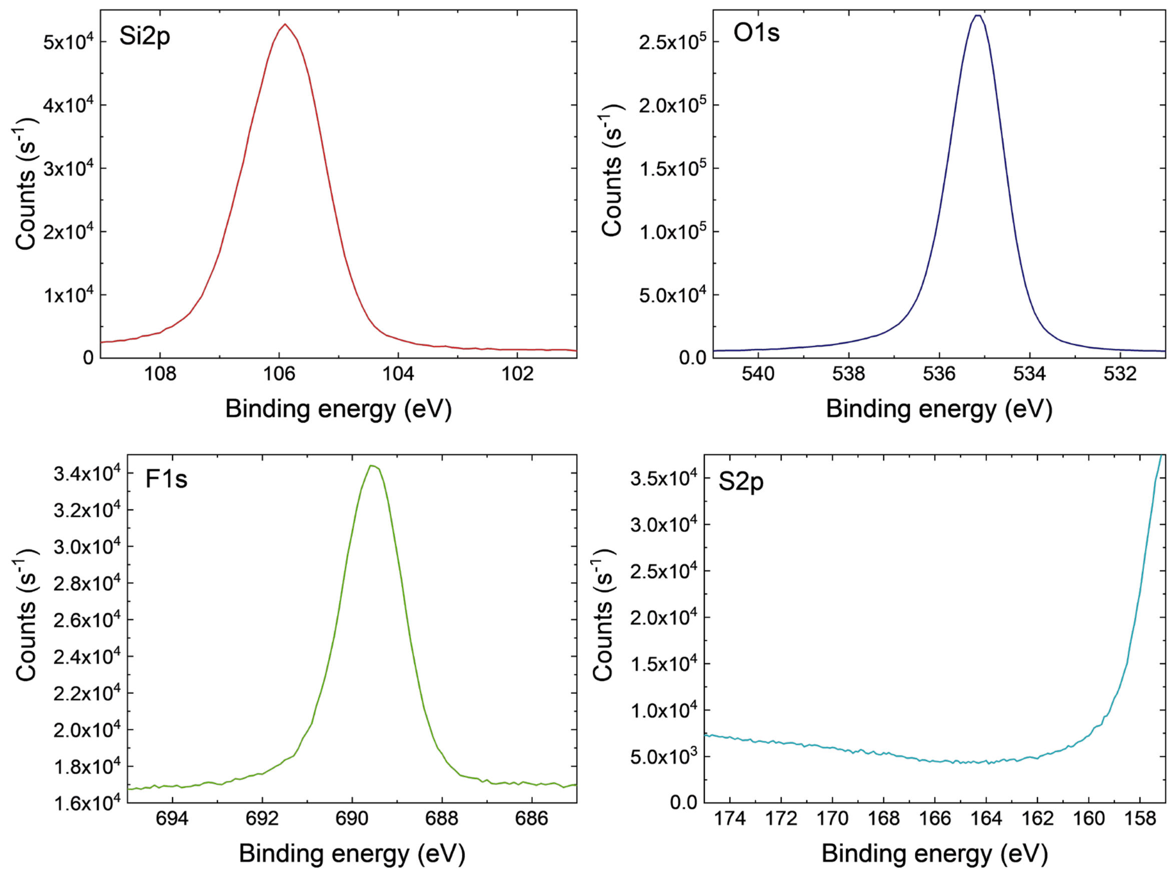

Figure S3: X-ray photoelectron spectra for Si, O, F and S of a SiO_2 surface exposed to 10 minutes of $SF_6/H_2/Ar$ plasma with $SF_6/(H_2+SF_6)$ = 0.24.

OH peak area integration:

The area of the OH-peak was determined for the spectra shown in Figure S3. By dividing the area for the peak after plasma exposure by the peak area after BDEAS adsorption, a ratio of 1.173 ± 0.008 was determined.

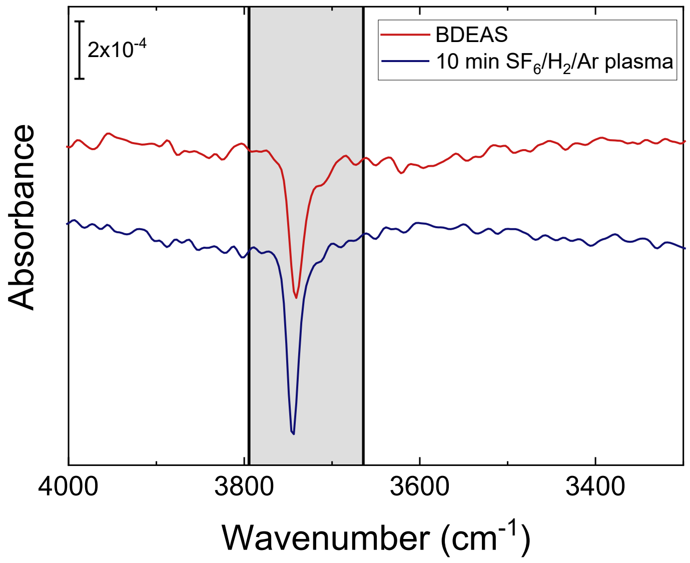

Figure S3: RAIRS spectra after BDEAS adsorption and a 600s $SF_6/H_2/Ar$ plasma with an $SF_6/(SF_6+H_2)$ ratio of ±0.23, with the spectrum before precursor or plasma exposure as the reference. To find the peak area, the area marked in gray has been integrated after a linear background has been removed from the relevant region of the spectrum.

Comparison of precursor adsorption on Al_2O_3:

Based on the RAIRS spectra shown in Figure S4, precursor adsorption on an as deposited and fluorinated Al_2O_3 were compared. Integrating both the OH and Si-H peaks and dividing the areas on the fluorinated substrate by those on the as-deposited substrate gave a ratio of 0.20 ± 0.01 and 0.71 ± 0.01 respectively. These ratios show that the OH consumption during precursor exposure is significantly more affected after plasma exposure than the amount of precursor that adsorbs, suggesting that the precursor can also adsorb on Al-F surface groups.

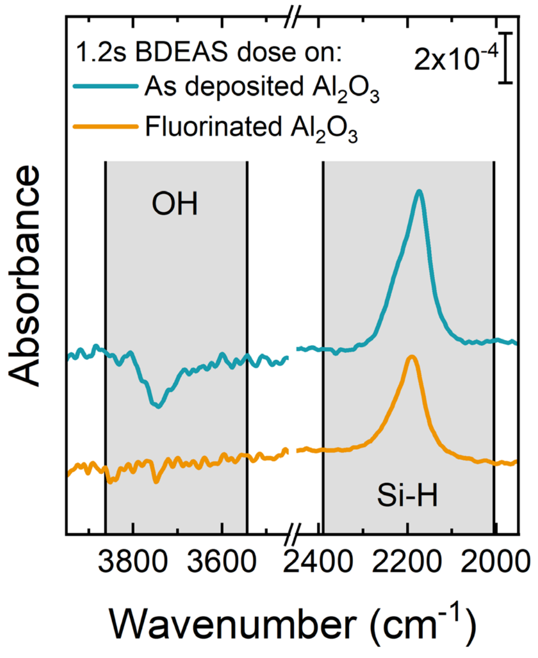

Figure S4: RAIRS spectra of BDEAS dosing on as deposited and plasma exposed Al_2O_3. The plasma had an $SF_6/(H_2+SF_6)$ flow ratio of ±0.23, with the spectrum before precursor dosing as the reference. The integrated areas are given in gray.

Simulation results for fluorination of Al_2O_3:

DFT simulations of Al_2O_3, shown in Figure S5, also confirmed that in the presence of HF, fluorination of the surface is energetically favorable. As explained above, the RAIRS data shows the removal of both Al-OH and Al-O-Al bonds. Therefore, OH- and Al-terminated Al_2O_3 slabs were used for simulations, and both resulted in the same F-terminated surface. In the case of the Al-terminated surface, the HF reacts and forms an Al-F bond, whereas the 3-coordinated oxygen (to Al) breaks 1-fold to form O-H bonds.

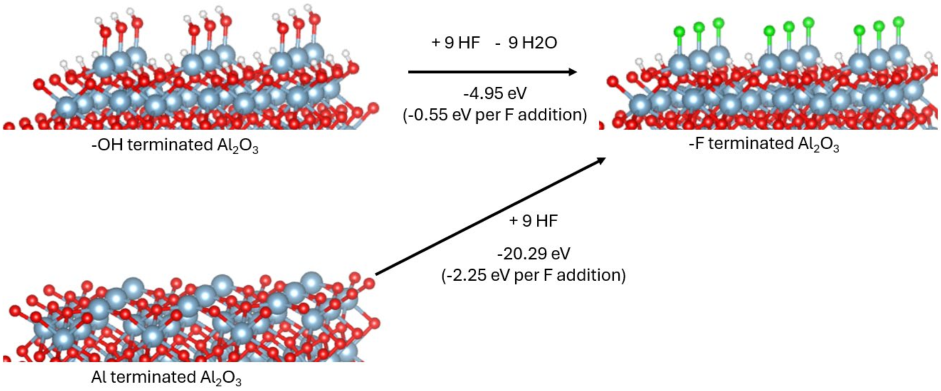

Figure S5: Density functional theory results of the fluorination of both -OH and Al terminated Al_2O_3.

TDMAT physisorption on hydroxylated and fluorinated Al_2O_3 and SiO_2:

The interaction energies between the TDMAT precursor and hydroxylated and fluorinated Al_2O_3 and SiO_2 were calculated to gain a better understanding of the mechanisms responsible for the selectivity of the ASD process. The results can be seen in Table S2. Both the physisorption energies and the distance between the precursor and the surface show that the physisorption of TDMAT is more favorable on Al_2O_3 than on SiO_2.

Table S2: Density functional theory results and the computed adsorption energies of TDMAT on hydroxylated and fluorinated Al_2O_3 and SiO_2. The dispersive ($\Delta E_{\mathrm{ads}}^{\mathrm{disp}}$) and electronic ($\Delta E_{ads}^{elec}$) components of the adsorption energy ($\Delta E_{\mathrm{ads}} = \Delta E_{ads}^{disp} + \Delta E_{ads}^{elec}$) are listed, together with the total adsorption energy, and the distance between the precursor atoms (Ti or N) and the surface atoms (O, H or F).

	**Al_2O_3-OH**	**SiO_2-OH**	**Al_2O_3-F**	**SiO_2-F**
**Configuration**	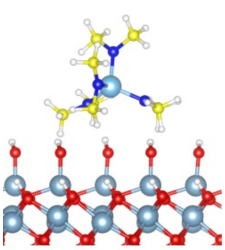	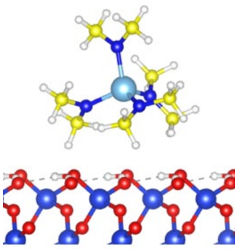	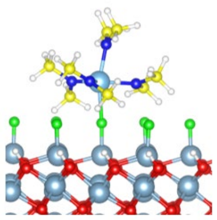	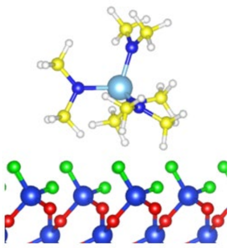
$\boldsymbol{\Delta E_{\mathrm{ads}}}$	-1.22 eV	-0.94 eV	-1.85 eV	-0.83 eV
$\boldsymbol{\Delta E_{\mathrm{ads}}^{\mathrm{disp}}}$	-0.75 eV	-0.71 eV	-0.96 eV	-0.56 eV
$\boldsymbol{\Delta E_{\mathrm{ads}}^{elec}}$	-0.47 eV	-0.23 eV	-0.89 eV	-0.27 eV
**Ti – O or Ti – F distance**	3.492 Å	4.272 Å	2.226 Å	3.690 Å
**N – H or N – F distance**	2.215 Å	3.418 Å	2.790 Å	3.208 Å

Mechanism for loss of selectivity:

Reducing the TDMAT purge time leads to a shorter nucleation delay. This indicates that some physisorbed TDMAT is likely staying on the non-growth area even after the purge, causing the onset of nucleation.

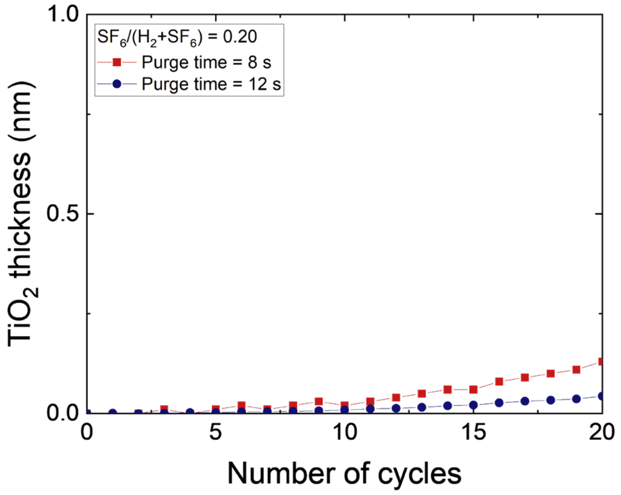

Figure S7: Nucleation curves of TiO_2 on SiO_2 after a 10 minute $SF_6/H_2/Ar$ plasma with a $SF_6/(SF_6+H_2)$ = 0.20, for different TDMAT purge times.

Precursor dosing after 10s of H_2O dosing, in pulses of 50ms, shows a negative peak in the OH region with a positive peak in the CH_3 stretch region, shown in Figure S6. This data suggests OH groups are formed during water exposure, which causes the loss of selectivity. Pumping the reactor after the TDMAT dosing shows some minor desorption of the precursor indicating that some of the precursors were physisorbed on the surface. This shows a second loss of selectivity mechanism.

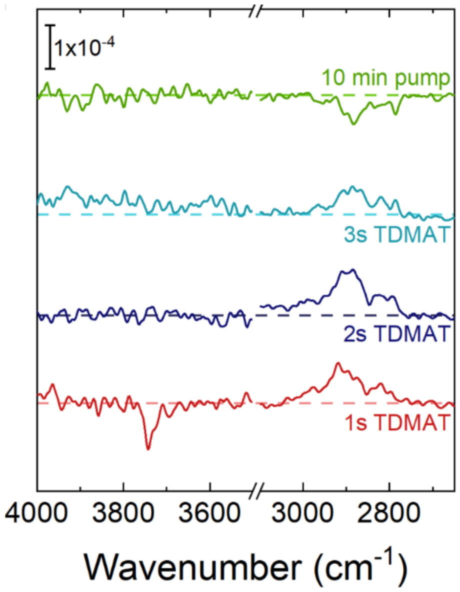

Figure S7: RAIRS difference spectra for TDMAT doses on a fluorinated SiO_2 surface after a total of 10 s of H_2O dosing. The spectrum after the water dosing was used as the reference for the first 1 s TDMAT. For each spectrum, the last spectrum before that precursor dose or pumping time was used as the reference.

References:

(1) Mackus, A. J. M.; Merkx, M. J. M.; Kessels, W. M. M. From the Bottom-Up: Toward Area-Selective Atomic Layer Deposition with High Selectivity. *Chemistry of Materials* **2019**, *31* (1), 2–12. https://doi.org/10.1021/acs.chemmater.8b03454.

(2) Parsons, G. N.; Clark, R. D. Area-Selective Deposition: Fundamentals, Applications, and Future Outlook. *Chemistry of Materials* **2020**, *32* (12), 4920–4953. https://doi.org/10.1021/acs.chemmater.0c00722.

(3) Merkx, M. J. M.; Sandoval, T. E.; Hausmann, D. M.; Kessels, W. M. M.; Mackus, A. J. M. Mechanism of Precursor Blocking by Acetylacetone Inhibitor Molecules during Area-Selective Atomic Layer Deposition of SiO_2. *Chemistry of Materials* **2020**, *32* (8), 3335–3345. https://doi.org/10.1021/acs.chemmater.9b02992.

(4) Merkx, M. J. M.; Tezsevin, I.; Yu, P.; Janssen, T.; Heinemans, R. H. G. M.; Lengers, R. J.; Chen, J. R.; Jezewski, C. J.; Clendenning, S. B.; Kessels, W. M. M.; Sandoval, T. E.; Mackus, A. J. M. In Situ Formation of Inhibitor Species through Catalytic Surface Reactions during Area-Selective Atomic Layer Deposition of TaN. *Journal of Chemical Physics* **2024**, *160* (20), 204701. https://doi.org/10.1063/5.0207496.

(5) Sik Yoo, W.; Swope, R.; Sparks, B.; Mordo, D. Comparison of C_2F_6 and FASi-4 as Fluorine Dopant Sources in Plasma Enhanced Chemical Vapor Deposited Fluorinated Silica Glass Films. *J Mater Res* **1997**, *12* (1), 70–74. https://doi.org/https://doi.org/10.1557/JMR.1997.0012.

(6) Campostrini, R.; Ischia, M.; Carturan, G.; Armelao, L. Sol-Gel Synthesis and Pyrolysis Study of Oxyfluoride Silica Gels. *J Solgel Sci Technol* **2002**, *23*, 107–117. https://doi.org/https://doi.org/10.1023/A:1013787115255.

(7) Lataste, E.; Demourgues, A.; Leclerc, H.; Goupil, J. M.; Vimont, A.; Durand, E.; Labrugère, C.; Benalla, H.; Tressaud, A. Access to Highly Fluorinated Silica by Direct F_2 Fluorination: Chemical Compositions and FTIR Investigations. *Journal of Physical Chemistry C* **2008**, *112* (29), 10943–10951. https://doi.org/10.1021/jp710790e.
